# Onset and mortality of Parkinson’s disease in relation to type II diabetes

**DOI:** 10.1007/s00415-022-11496-y

**Published:** 2022-11-27

**Authors:** Gianni Pezzoli, Emanuele Cereda, Paolo Amami, Santo Colosimo, Michela Barichella, Giorgio Sacilotto, Anna Zecchinelli, Michela Zini, Valentina Ferri, Carlotta Bolliri, Daniela Calandrella, Maria Grazia Bonelli, Viviana Cereda, Elisa Reali, Serena Caronni, Erica Cassani, Margherita Canesi, Francesca del Sorbo, Paola Soliveri, Luigi Zecca, Catherine Klersy, Roberto Cilia, Ioannis U. Isaias

**Affiliations:** 1 Parkinson Institute Milan, ASST-Pini-CTO, Via Bignami 1, Milan, Italy; 2 Fondazione Grigioni per il Morbo di Parkinson, Milan, Italy; 3 Clinical Nutrition and Dietetics Unit, Fondazione IRCCS Policlinico San Matteo, Viale Golgi 19, 27100 Pavia, Italy; 4 University of Milan, Specialization School in Nutrition Science, Milan, Italy; 5 Clinical Nutrition Unit, ASST-Pini-CTO, Milan, Italy; 6 Programming and Grant Offices (UPGO), Italian National Research Council (CNR), Rome, Italy; 7 Dietetic and Clinical Nutrition Unit, ASST-Fatebenefratelli-Sacco, University Hospital, Milan, Italy; 8 Department of Parkinson’s Disease, Movement Disorders and Brain Injury Rehabilitation, “Moriggia-Pelascini” General Hospital, Como, Italy; 9 Institute of Biomedical Technologies, National Research Council of Italy, Segrate, Milan Italy; 10 Unit of Clinical Epidemiology and Biometry, Fondazione IRCCS Policlinico San Matteo, Pavia, Italy; 11 Parkinson and Movement Disorders Unit, Fondazione IRCCS Istituto Neurologico Carlo Besta, Milan, Italy; 12 Department of Neurology, University Hospital of Würzburg and Julius Maximilian University of Würzburg, Würzburg, Germany

**Keywords:** Parkinson’s disease, Diabetes, Age at onset, Prevalence, Mortality

## Abstract

**Objectives:**

There is growing evidence that Parkinson’s disease and diabetes are partially related diseases; however, the association between the two, and the impact of specific treatments, are still unclear. We evaluated the effect of T2D and antidiabetic treatment on age at PD onset and on all-cause mortality.

**Research design and methods:**

The standardized rate of T2D was calculated for PD patients using the direct method and compared with subjects with essential tremor (ET) and the general Italian population. Age at onset and survival were also compared between patients without T2D (PD-noT2D), patients who developed T2D before PD onset (PD-preT2D) and patients who developed T2D after PD onset (PD-postT2D).

**Results:**

We designed a retrospective and prospective study. The T2D standardized ratio of PD (*N* = 8380) and ET (*N* = 1032) patients was 3.8% and 6.1%, respectively, while in the Italian general population, the overall prevalence was 5.3%. In PD-preT2D patients, on antidiabetic treatment, the onset of PD was associated with a + 6.2 year delay (*p* < 0.001) while no difference was observed in PD-postT2D. Occurrence of T2D before PD onset negatively affected prognosis (adjusted hazard ratio = 1.64 [95% CI 1.33–2.02]; *p* < 0.001), while no effect on survival was found in PD-postT2D subjects (hazard ratio = 0.86, [95% CI 0.53–1.39]; *p* = 0.54).

**Conclusions:**

T2D, treated with any antidiabetic therapy before PD, is associated with a delay in its onset. Duration of diabetes increases mortality in PD-preT2D, but not in PD-postT2D. These findings prompt further studies on antidiabetic drugs as a potential disease-modifying therapy for PD.

**Supplementary Information:**

The online version contains supplementary material available at 10.1007/s00415-022-11496-y.

## Introduction

Parkinson’s disease (PD) and related syndromes constitute the second most common group of neurodegenerative conditions. It has been noted that from 1990 to 2015 the number of people with PD doubled to over 6 million and this figure is predicted to double again to over 12 million by 2040, mainly because of the increasing average age of the population [1, 2]. The increasing prevalence of PD is of concern due to the huge burden this disease places on patients, caregivers and public healthcare budgets.

PD is mainly managed symptomatically using dopaminergic drugs, with an excellent response in the early years of the disease. Disease progression demonstrates that motor symptoms re-emerge after 5–7 years of relative well-being [3]. Efforts to find disease-modifying agents have not met with success, and none of the randomized trials have shown convincing effects of putative agents on PD progression [4].

PD and type II diabetes (T2D) have been established to share underlying mechanisms, including mitochondrial dysfunction, under-expression of the transcriptional regulator PPARγ coactivator 1α, oxidative stress, and inflammation [5]. Interestingly, a significant decrease in ^11^C-donepezil (a high-affinity ligand for acetylcholinesterase) signal was demonstrated in the pancreas of PD patients [6], as in patients with type I diabetes [7], indicating parasympathetic denervation in PD. Furthermore, cytoplasmic phosphorylated alpha-synuclein deposits were found in the pancreatic beta-cells of subjects with PD and T2D [8, 9]. Insulin resistance was found in 60% of PD patients, 30% of whom developed glucose intolerance [10], and increased levels of alpha-synuclein negatively affect glucose-stimulated insulin secretion in pancreatic beta-cells of the *Rip/Snca* transgenic mice model [11].

There are several epidemiologic studies connecting PD and T2D; however, there is no agreement on the risk of diabetic patients to develop PD: reviews and meta-analyses have reached opposite conclusions [12–16]. Concordant are instead the clinical studies, with cross-sectional observations suggesting that T2D is associated with a more aggressive PD phenotype and an enhanced progression of PD symptoms [17–19]. A recent prospective study has reported a comparable, accelerated progression associated with good or poor glycemic control, respectively [20]. It seems that T2D, when treated, has no detrimental effect on PD clinical phenotype. Further evidence suggests that some antidiabetic drugs may protect against neurodegeneration. A randomized controlled trial of exenatide—a glucagon-like peptide-1 (GLP-1) receptor agonist used to treat T2D—found that the drug had positive effects on practically all off-medication motor features, which persisted even beyond the period of drug exposure [21]. It has also been observed that the incidence of PD in patients with T2D was significantly lower in those given GLP-1 receptor agonists or dipeptidyl peptidase 4 (DPP4) inhibitors compared with individuals prescribed any other oral combination therapy for diabetes [22–24]. Additional evidence comes from the use of bromocriptine, a dopamine receptor agonist, as useful adjunctive agent in the management of T2D. Both bromocriptine and dopamine mediate in vivo tissue glucose uptake in rodents [25]. Furthermore, in the 6-OHDA-induced PD mouse model, metformin itself partially ameliorates akinetic-rigid symptoms [26].

The aim of the present study was to investigate the relationship between PD and T2D by observing a large cohort of consecutive patients presenting at the Parkinson Institute Milan (ASST-Pini-CTO) over a 10-year period. Presently, there are no comparative data on the onset and evolution of PD in subjects who developed it while taking or not taking antidiabetic therapy. We evaluated age at onset of PD and estimated the prevalence of T2D in PD compared with the general population and subjects with essential tremor (ET). We also investigated the effects of T2D occurrence in relation to PD onset on all-cause mortality.

## Patients and methods

### Patients

**Fig. 1:**
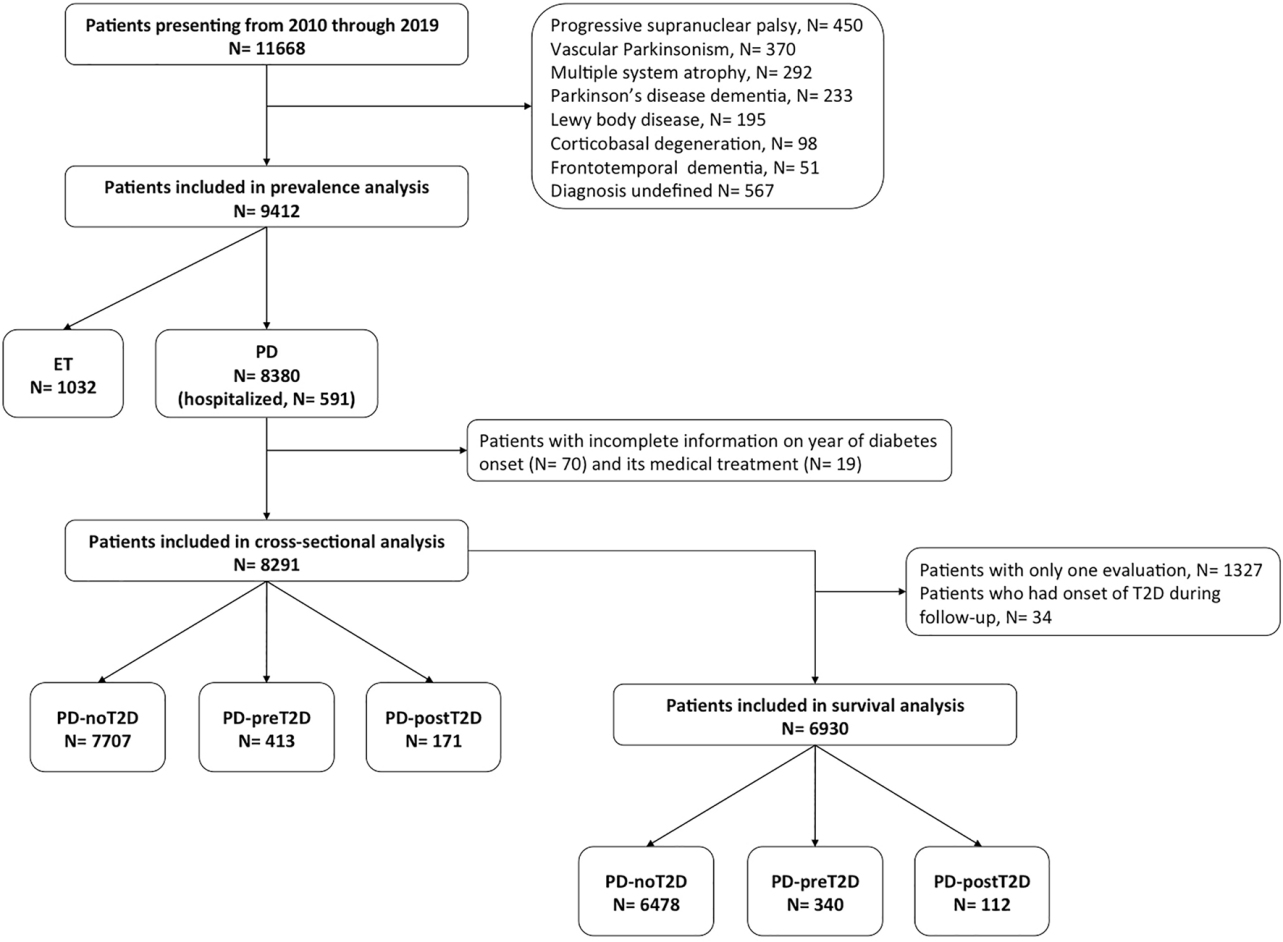
Flowchart of patient selection. Abbreviations: *PD-noT2D* PD patients without T2D, *PD-preT2D* patients with T2D occurrence before PD onset, *PD-postT2D* patients with T2D occurrence after PD onset

For this retrospective study using prospectively collected data, the database of the Parkinson Institute Milan (ASST-Pini-CTO), including consecutive patients followed at our clinical centre over a period of 10 years (from January 2010 to December 2019), was queried. Patients with an established diagnosis of PD and ET were included in the study, while patients with other neurodegenerative diseases, vascular disease or undefined diagnosis as well as patients with type I diabetes were excluded (Fig. 1).

The diagnosis of PD or ET was established by a movement disorder specialist, based on established diagnostic criteria for PD [27, 28] and ET [29], with the support of neuroimaging findings (brain magnetic resonance imaging, dopamine transporter single photon emission computed tomography, 18F-fluorodeoxyglucose positron-emission tomography, etc.).

### Clinical evaluation

At baseline (the first visit at the Parkinson Institute Milan, ASST-Pini-CTO), all patients underwent a thorough neurological examination and had self-completed a comprehensive questionnaire including more than 100 items covering a broad range of aspects (e.g., family history, life-style, past medical history, occupational history, detailed history of the neurodegenerative disease, previous and present medication) that was subsequently reviewed by a neurologist. The same questionnaire was updated during each follow-up visit. Patients who reported the occurrence of T2D were selected for a further health interview, with caregiver support if necessary, to confirm their history of diabetes. Information regarding the onset of T2D and current antidiabetic medical treatment was further confirmed through prescriptions from the family doctor, referral diabetologist or by accessing the health data register.

All patients were visited at the outpatient clinic and a subgroup was hospitalized for additional diagnostic and/or therapeutic purposes (herein identified as “inpatients” group). These patients underwent blood glucose and glycosylated hemoglobin after overnight fasting according to consensus criteria [30]. This subgroup was used as an internal control to assess the accuracy of self-reported data on diabetes (onset, therapy, etc.).

The study endpoints were the prevalence of diabetes at the last available visit, age at onset of PD and all-cause mortality. Specifically, age at onset was defined as the age when the first cardinal motor symptoms appeared, as reported by the patient with the aid of a family member or caregiver when necessary. Survival was defined as the time that had elapsed (in years) between the first evaluation at the Parkinson Institute Milan and the date of death or the date of last contact (censoring). Vital status was ascertained by means of active follow-up (in-office visits, telephone or mail enquiries to participants or proxy respondents and linkage to municipal registries) up to February 2020.

### Statistical analysis

#### Prevalence

Prevalence of T2D was calculated for PD outpatients, PD inpatients and ET patients at the date of last contact. The direct method was used to standardize raw prevalence based on the most recent data on gender and age distribution of the Italian population (from the 2016 survey by the Italian National Institute of Statistics, https://www.istat.it/it/archivio/202600).

#### Age at PD onset

At the end of follow-up, PD patients were grouped based on T2D occurrence in relation to PD onset as follows: patients who never developed T2D (PD-noT2D), patients who had T2D before PD onset (PD-preT2D) and patients in whom T2D occurred after PD onset (PD-postT2D, Fig. 1). We compared demographic and clinical data between these three groups.

#### Survival

Survival time was defined as the number of years that had elapsed from the first visit to the outcome or to the date of last contact. Outcome was the patient’s death.

Continuous variables were reported as means and standard deviations (SD), categorical variables as frequencies and percentages (%). The normal distribution of continuous variables was checked with the Kolmogorov–Smirnov and Shapiro–Wilk tests. Between-group comparisons were performed using the Chi-squared test, one-way ANOVA or the Kruskal–Wallis test based on the nature and distribution of variables. Post hoc comparisons were performed using the Bonferroni correction. The effect of the duration of T2D treatment on age of PD onset was assessed by a multivariable linear regression analysis. The log rank test was used to compare survival curves between the three groups. The Cox proportional hazards regression model was used to investigate the association between the occurrence of T2D in relation to PD onset on survival time (PD was considered the reference group); covariates included gender, age at baseline, PD duration at baseline and comorbidities (heart disease, hypertension, stroke, and tumor). For all analyses, the statistical level was set at *p* < 0.05. All statistical analyses were performed using the SPSS 25 software package.

### Ethics approval

The Ethics Committee of Fondazione IRCCS Cà Granda Ospedale Maggiore Policlinico, Milano, Regione Lombardia-Italy approved the study “dated favorable opinion 18.03.2020/opinion 170_2020bis”. The study was performed in accordance with the ethical principles of the Declaration of Helsinki and its later amendments. All subjects provided written informed consent to participate in the study.

### Data sharing

Data sharing is available upon reasonable request to the corresponding author who will evaluate on a case-by-case basis.

## Results

From January 2010 through December 2019, 11,668 patients presented at the Parkinson Institute Milan (ASST-Pini-CTO), of whom 8380 and 1032 had an established diagnosis of PD and ET, respectively (Fig. 1). Five hundred and ninety-one PD patients were hospitalized for diagnostic or therapeutic purposes (Fig. 1) and no undiagnosed case of T2D was identified. The antiparkinsonian and antidiabetic therapies are listed in one of our previous works [17]. The percentage distribution of antidiabetic drugs at the last visit for PD-preT2D and PD-postT2D is further detailed in Supplementary Table 1.

### Prevalence

At the last follow-up visit, a total of 673 (8.4%) PD patients and 150 (14.5%) ET patients had T2D. A comparison of the standardized prevalence of T2D identified a lower frequency in PD patients (3.8%) than in the Italian general population (5.3%) and ET patients (6.1%, Table 1). Moreover, the standardized rate of T2D was lower in PD patients aged ≥ 65 compared with the Italian population and ET patients (Table 1). The standardized rate of T2D in PD inpatients, who underwent blood glucose and glycosylated haemoglobin evaluation, was comparable to the rate in PD outpatients (Table 1).

**Table 1:** T2D prevalence and standardized prevalence in PD outpatients, hospitalized PD patients and ET patients

	Total PD patients *N* = 8380	PD outpatients *N* = 7789	PD inpatients *N* = 591	ET patients *N* = 1032	Italian population *N* = 60,433,962
No of patients with T2D	673 (8%)	629 (8.1%)	44 (7.4%)	150 (14.5%)	3,203,000
T2D standardized rate	3.8%	3.91%	3.14%	6.08%	5.3%
T2D standardized rate (≥ 65)	9.01%	8.97%	10.19%	16.44%	16.4%

The raw prevalence rates were standardized based on gender and age distribution of the Italian population using the direct method

### Age at PD onset

PD patients with no reliable information regarding the date of T2D diagnosis and those not taking antidiabetic medication were excluded from the study (Fig. 1). T2D occurred before the onset of PD in 413 patients, while 171 patients developed T2D after PD onset. Demographic and clinical features of PD patients based on T2D occurrence in relation to PD onset are reported in Table 2. Age at PD onset was approximately 67 years in PD-preT2D patients, a higher mean onset age of 7 years compared with both PD-postT2D and PD patients without T2D (*p* < 0.001). This difference was maintained after adjusting for gender, coffee consumption and smoking: compared with PD-noT2D, patients with T2D prior to the onset of PD were older (a mean difference of 6 years, 95% CI 3.3–8.5, *p* < 0.001), while those who developed T2D after PD had a comparable age at PD onset (*p* = 0.99). A multivariate regression analysis showed that age at PD onset was related to the duration of T2D treatment (*p* < 0.001), as patients who had been treated for T2D over a longer time period were older at PD onset (Supplementary Table 2). PD onset in patients who had had T2D for seven years or less was delayed by an average of 4.8 years, while patients with a T2D duration of over 7 years displayed a mean delay of 5.7 years compared with patients without T2D before PD onset (Supplementary Table 3). We also evaluated the effect that metformin treatment had on age of PD onset compared with other antidiabetic treatments, but we found no difference (*p* = 0.525) (Supplementary Table 4). BMI calculated during the last visit was higher both in the PD-preT2D and PD-postT2D groups compared with PD patients without diabetes (*p* < 0.001). The occurrence of heart diseases (*p* < 0.001) and hypertension (*p* < 0.001) was higher in both groups of PD patients with diabetes compared to non-diabetic PD patients, while the occurrence of strokes was higher only in PD-preT2D patients (*p* = 0.002). There was no difference between groups regarding occurrence of tumors (*p* = 0.099). Consumption of coffee and smoking were comparable between the three groups.

**Table 2:** Comparison of PD-preT2D, PD-postT2D and PD-noT2D patients at the last contact (total number of patients = 8291)

	PD-noT2D *N* = 7707	PD-preT2D *N* = 413	PD-postT2D *N* = 171	*p* value
Gender (f%)	3258 (42.3%)	129 (31.2%)	68 (39.8%)	< 0.001
Age at PD onset (years)	60.71 (± 11.01)	66.87 (± 8.5)	60.58 (± 10.88)	< 0.001^a^
Age at T2D onset (years)	–	56.71 (± 10.96)	66.11 (± 9.82)	< 0.001
Age at last contact (years)	72.52 (± 10.33)	75.35 (± 8.02)	73.74 (± 8.85)	< 0.001^b^
PD duration at last contact (years)	11.81 (± 7.41)	8.47 (± 5.41)	13.16 (± 7.28)	< 0.001^c^
T2D duration at last contact (years)	–	18.63 (± 9.98)	7.74 (± 5.94)	< 0.001
BMI at last contact	25.22 (± 4.04)	27.28 (± 4.31)	27.61 (± 4.66)	< 0.001^d^
Heart diseases (occurrence%)	1433 (18.6%)	113 (27.4%)	46 (26.9%)	< 0.001
Hypertension (occurrence%)	2824 (36.6%)	257 (62.2%)	93 (54.4%)	< 0.001
Stroke (occurrence%)	289 (3.7%)	30 (7.3%)	8 (4.7%)	0.002
Tumor (occurrence%)	844 (11%)	54 (13.1%)	26 (15.2%)	0.099

For categorical variables a Chi-squared test was used

For continuous variables the Mann–Whitney test was used for a comparison between two groups and the Kruskal–Wallis test was used for a comparison among three groups; for post hoc comparisons the Bonferroni correction was used: ^a^PD-preT2D ≠ PD-postT2D = PD-noT2D; ^b^PD-preT2D ≠ PD-noT2D; ^c^PD-preT2D ≠ PD-post-T2D ≠ PD-noT2D; ^d^PD-noT2D ≠ PD-preT2D = PD-postT2D

### Survival

**Fig. 2:**
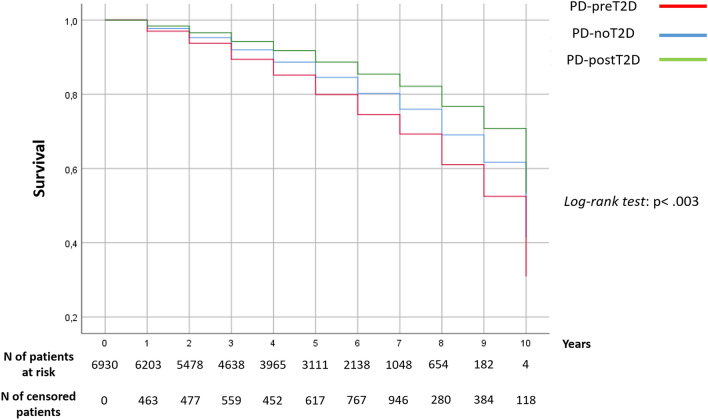
Kaplan–Meier mortality curves of PD-preT2D, PD-postT2D and PD-noT2D patients (log-rank test: *χ*(2) = 11.459, *p* < 0.003). Abbreviations: *PD-noT2D* PD patients without T2D, *PD-preT2D* patients with T2D occurrence before PD onset, *PD-postT2D* patients with T2D occurrence after PD onset

Survival analysis included a total of 6,930 PD patients, as 1,327 patients were excluded since they did not attend follow-up and 34 patients experienced T2D onset during the observation period (Fig. 1). The baseline features of the three groups are shown in Table 3. After a median follow-up of 60 months [IQR 36–84], 1877 patients (27.1%) had died. PD-preT2D patients experienced higher mortality rates: 1,742 (26.9%) deaths occurred in patients without T2D, 111 (32.6%) in PD-preT2D patients and 24 (21.4%) in PD-postT2D patients (*p* < 0.003; Fig. 2). Cox hazards analysis showed an association between mortality and the occurrence of T2D before PD onset, as this group of patients had an increased hazard ratio compared to PD patients without diabetes (fully adjusted *HR* = 1.64 [95% CI 1.33–2.02]; *p* < 0.001), while patients who developed T2D after PD onset had a hazard ratio comparable to patients without diabetes (*HR* = 0.86, [95% CI 0.53–1.39]; *p* = 0.54) (Supplementary Table 5). There was no difference in mortality when we compared PD patients with T2D treated with metformin or other antidiabetic drugs (*p* = 0.265).

**Table 3:** Baseline features of PD-preT2D, PD-postT2D and PD-noT2D patients who participated in survival analysis (total number of patients = 6930)

	PD-noT2D *N* = 6478	PD-preT2D *N* = 340	PD-postT2D *N* = 112	*p* value
Gender (f%)	2756 (42.5%)	106 (31.2%)	43 (38.4%)	< 0.001
Age at baseline (years)	68.1 (± 10.3)	71.4 (± 8)	69.5 (± 8.8)	< 0.001^a^
PD duration (years)	7.8 (± 6.7)	4.8 (± 4.2)	8.1 (± 5.9)	< 0.001^b^
T2D duration (years)	–	14.4 (± 9.5)	4.5 (± 5.5)	< 0.001
Follow-up duration (months)	59.6 (± 29.7)	55 (± 30.6)	60 (± 33.4)	0.017 ^a^
Heart diseases (occurrence%)	1217 (18.8%)	93 (27.4%)	32 (28.6%)	< 0.001
Hypertension (occurrence%)	2405 (37.1%)	212 (62.4%)	64 (57.1%)	< 0.001
Stroke (occurrence%)	261 (4%)	23 (6.8%)	4 (3.6%)	0.047
Tumor (occurrence%)	706 (10.9%)	48 (14.1%)	14 (12.5%)	167

For categorical variables, the Chi-squared test was used

For continuous variables the Mann–Whitney test was used for a comparison between two groups and the Kruskal–Wallis test for a comparison among three groups; for post hoc comparisons Bonferroni’s correction was used: ^a^PD-preT2D ≠ PD-noT2D; ^b^PD-preT2D ≠ PD-postT2D = PD-noT2D

## Discussion

This work stems from a previous case–control design study [17], which showed a worsening trend in motor symptoms in PD-preT2D when compared with PD-noT2D, a finding later confirmed by other authors [31]. Herein, we present data on age at onset in PD-preT2D and PD-postT2D patients, the prevalence of diabetes in PD, and mortality rates across the various groups. Clinical data were collected in a single third-level Italian centre (Parkinson Institute Milan, ASST-Pini-CTO, Milan, Italy). This might be a limitation of the current study, as complex patients are more easily accessed in a third-level centre. Less demanding patients are followed by general neurologists or even by their general practitioners. Besides, data generalizability might be affected by the ethnic homogeneity of our cohort (only 24 non-Caucasians patients). Conversely, there are positive aspects: nine highly experienced neurologists on movement disorders work at the Parkinson Institute Milan providing consistent and comparable follow-up evaluations; diagnoses are more conclusive after a few years of illness.

The result is a retrospective cohort study regarding the onset of PD and T2D, prospective as regards mortality. Collecting mortality data were not a major issue because in addition to family members, the municipality of residence and place of birth of all the patients in the database were consulted, if deemed necessary. Nor was it difficult to establish the age of onset of PD as only motor symptoms were considered. The average age of all PD patients at onset is very similar to those presented by other authors [32]. It was more difficult to establish with relative certainty the onset of diabetes and the beginning of antidiabetic therapy because the disease itself is paucisymptomatic and patients tend to lose long-standing clinical documentation over the years. Therefore, potential bias associated with self-reporting is also acknowledged taking into account that not all patients underwent full screening procedures for diabetes, although in the subgroup of inpatients—used as internal control—no undiagnosed case of T2D was identified. For this reason, we excluded 70 diabetic patients who were unable to document the disease’s onset with any certainty (Fig. 1), and present only the antidiabetic therapy related to the last follow-up visit. We also recognize that glycemic control for each diabetic patient in this study was not available, except for those patients who were hospitalized, in which, however, it was sufficiently controlled but we cannot exclude that these data, along with information on organ damage, would have improved our analyses further. Noteworthy, polymorbid patients are likely to undergo increased medical surveillance and this would have strengthened the reliability of the data analysed herein but we cannot also exclude that some signs of PD might have been interpreted as signs of DM by general practitioners. In Italy, diabetic patients are entitled to free prescriptions, which allows us to exclude bias related to economic status. Besides, the new GLP-1 receptor agonists are provided at no cost only to diabetic patients intolerant to metformin: only few patients are treated with these drugs. As regards gender, the characteristics of the cohort are similar to those reported by other databases [33] with a moderate male prevalence in the total parkinsonian population and even more marked in the diabetic group (Table 2) [34].

This work focuses on age of onset of PD in the non-diabetic population, compared with that of a cohort of diabetic parkinsonian patients. The data are solid with a large sample size. Diabetic patients on any form of antidiabetic therapy were found to report development of PD more than 6 years later than non-diabetic subjects or subjects who go on to develop diabetes after the onset of PD. Until now, there are no known treatments or pathologies that can cause a delay in the onset of PD of this magnitude. The literature already cites studies reporting that diabetic subjects have a delayed onset of PD of the same magnitude reported by Ou and coll [20], but the data have never been evaluated in depth by dividing diabetic patients who developed the disease before or after the onset of PD. This allows us to observe that the age of onset of PD in PD-postT2D patients is identical to that in non-diabetic parkinsonian subjects (Table 2).

Delayed onset of PD by about 6 years in PD-preT2D corresponds to approximately 30% of the average life expectancy of a parkinsonian patient. Therefore, it is reasonable to expect a reduction in the prevalence of diabetes in PD, as the delayed onset means that a certain percentage of diabetic patients will not live long enough to develop PD. Compared with official Italian data from 2016, of those who “claim to have diabetes” the prevalence in our groups is about − 30% among all parkinsonian patients. In this regard, data in the literature are rather controversial [14–16]. In particular, out of 18 studies considered in the most recent review by Camargo Maluf and coll [35], the risk of developing PD in patients with T2D was increased in nine studies and decreased or unrelated in nine. Interestingly, the studies that observed a reduced risk of developing diabetes in PD enrolled fewer patients but had better characterized case series. In view of this, we tried to combine a large-sample cohort study with diagnostic accuracy carried out over an average 5-years period, confirmed in virtually all patients by instrumental examinations: MRI or CT scan, DaTScan (i.e., SPECT with Ioflupane) and PET with fluorodeoxyglucose (FDG), and less frequently with a CSF examination. In addition, subjects from the three groups were enrolled in the study between five and nine years (Table 3) after the onset of PD, when symptoms and response to levodopa are generally sufficiently clear and the diagnostic uncertainties of the initial period are overcome. With these methods, ET, a highly frequent disorder, and parkinsonisms with a prevalent vascular component, or other movement disorders of uncertain diagnosis, were differentiated. Mortality data suggested a significant, increased risk in subjects with pre-diabetes compared to subjects without diabetes or with post-diabetes. These results are in line with clinical data available in the literature, especially in PD-preT2D, indicating a more severe and less responsive form of PD than in age- and sex-matched, non-diabetic PD patients [17, 18, 24].

Late onset of PD and early mortality in PD-preT2D subjects can therefore account for the reduced prevalence of diabetes in PD that we observed. It could be argued that treatment of diabetes extends the life of those affected to an age when they (can) develop PD. In addition, people with T2D without PD have higher mortality rates and are likely to die at earlier age than the general population [36].

In the group of patients with PD-preT2D (approximately 20 years of diabetes at death), the risk of death was higher, despite the fact they had only a 12-years history of PD compared to PD-noT2D subjects with an 18-years history of the disease. In the PD-postT2D group, however, with a 10-years history of diabetes before death, no increased risk of mortality was found. We can therefore speculate that diabetes treated with antidiabetic drugs may be associated with late onset of PD. During the first ten years of diabetes, the risk of mortality for PD-postT2D patients is not increased when compared with patients with PD only. This cohort study confirms what many studies now suggest, as well as the opinion of experts [37] in an increasing number of reports in the medical literature, namely, that antidiabetic drugs could have a beneficial effect when used in PD, alpha-synucleinopathies and other neurodegenerative diseases. This hypothesis is difficult to establish and does not find a causal basis in this work. Still, the results presented suggest a correlation of great interest for its potential preventive impact and for the study of novel disease-modifying strategies. In this study, due to limitations in the number of cases of T2D we could not demonstrate any difference between metformin and other antidiabetic agents for delayed disease onset and mortality, but current research mainly focuses on drugs that do not reduce blood sugar and thus can be tested as neurotrophic drugs even in non-diabetic patients. Diabetes alone, with or without drug treatment, could theoretically be fully or partly responsible for this phenomenon. We are unable to report any data in this regard, as only 19 patients in our group are on diet therapy alone. We are also unable to state that this phenomenon is dopaminergic per se as we do not have systematic data such as DaTScan imaging in this patient group. Even if the phenomenon of delayed onset of PD is not related to antidiabetic treatment but is a feature of diabetes itself, it would still be interesting to understand the pathophysiological basis of this phenomenon with future studies. We do believe there are very interesting premises for considering a study addressing the use of antidiabetic drugs at least in those subjects who present what is now considered a preclinical phase of PD, characterized by constipation, hypo/anosmia, REM behaviour-disorder that can occur up to 10–15 years before the onset of motor symptoms [38].

Being able to positively modify the course of PD, even in a prodromal phase by 30%, would make the management of this disease much less complex for neurologists and less painful for patients and caregivers. Controlled longitudinal studies are needed to confirm these findings in PD and other neurodegenerative diseases. However, it is objectively difficult to carry out a controlled study able to demonstrate that antidiabetic drugs have the ability to partially prevent PD.

## Supplementary Information

Below is the link to the electronic supplementary material.

Supplementary file1 (DOCX 36 KB)

## Data Availability

The data that support the findings of this study are available from the corresponding author upon reasonable request and subjected to approval by the local Ethics Committee.

## Abbreviations

ET: Essential tremor
PD: Parkinson’s disease
T2D: Type II diabetes
