# Supplementary material for: Onset and mortality of Parkinson’s disease in relation to type II diabetes
Source: J Neurol. 2022 Nov 27;270(3):1564–72. doi: 10.1007/s00415-022-11496-y (PMC9971073; doi:10.1007/s00415-022-11496-y)
Supplement: Supplementary file 1 — Supplementary file1 (DOCX 36 KB) [file 415_2022_11496_MOESM1_ESM.docx]

***Onset and mortality of Parkinson’s disease in relation to type-II diabetes***

**Running title:** Type-II diabetes and Parkinson’s disease

Gianni Pezzoli MD^1,2^, Roberto Cilia MD^3^, Paolo Amami PsyD^1,2^, Santo Colosimo MD^4,5^, Michela Barichella MD^5^, Giorgio Sacilotto MD^1^, Anna Zecchinelli MD^1^, Michela Zini MD^1^, Valentina Ferri MD^2,5^, Carlotta Bolliri ScD^2,5^, Daniela Calandrella MD^1,2^ Maria Grazia Bonelli PhD^6^, Viviana Cereda PsyD ^1,2^, Elisa Reali PsyD ^1,2^, Serena Caronni ScD^2,5^, Erica Cassani MD^2,5,7^, Margherita Canesi MD^1,8^, Francesca del Sorbo MD^1^, Paola Soliveri MD^1^, Luigi Zecca MD^9^, Catherine Klersy MD^10^, Emanuele Cereda MD*^11^§ and Ioannis U. Isaias MD*^12^

1. Parkinson Institute Milan, ASST-Pini-CTO, via Bignami 1, Milan, Italy
2. Fondazione Grigioni per il Morbo di Parkinson, Milan, Italy
3. Fondazione IRCCS Istituto Neurologico Carlo Besta, Parkinson and Movement Disorders Unit, Milan, Italy
4. University of Milan, Specialization School in Nutrition Science, Milan, Italy
5. Clinical Nutrition Unit, ASST-Pini-CTO, Milan, Italy
6. Programming and Grant Offices (UPGO), Italian National Research Council (CNR), Roma, Italy
7. Dietetic & Clinical Nutrition Unit, ASST-Fatebenefratelli-Sacco, University Hospital, Milan, Italy
8. Department of Parkinson's Disease, Movement Disorders & Brain Injury Rehabilitation, “Moriggia-Pelascini” General Hospital, Como, Italy.
9. Institute of Biomedical Technologies, National Research Council of Italy, Segrate, Milan, Italy.
10. Unit of Clinical Epidemiology & Biometry, Fondazione IRCCS Policlinico San Matteo, Pavia, Italy
11. Clinical Nutrition and Dietetics Unit, Fondazione IRCCS Policlinico San Matteo, Pavia, Italy
12. Department of Neurology, University Hospital of Würzburg and Julius Maximilian University of Würzburg, Würzburg, Germany.

* These authors contributed equally to the study.

§ **Contact information for corresponding author:**

Emanuele Cereda MD, PhD;

Clinical Nutrition and Dietetics Unit, Fondazione IRCCS Policlinico San Matteo,

Viale Golgi 19, 27100 Pavia, Italy;

Tel: +39 0382 501615, Fax: + 39 0382 502801,

E-mail: [e.cereda@smatteo.pv.it](mailto:e.cereda@smatteo.pv.it).

**Supplementary table 1.** Antidiabetic medical treatment of PD-preT2D and PD-postT2D.

| Medical treatment | All PD patients with T2D | PD-preT2D | PD-postT2D |
| --- | --- | --- | --- |
| With metformin | N= 360 | N= 269 | N= 91 |
| Alone | 214 (59.4%) | 156 (57.9%) | 58 (63.7%) |
| + Sulfonylurea | 44 (12.2%) | 34 (12.6%) | 10 (10.9%) |
| + Glinide | 8 (2.2%) | 6 (2.2%) | 2 (2.1%) |
| + Thiazolidinedione | 11 (3%) | 10 (3.7%) | 1 (1.1%) |
| + Alpha-glucosidase inhibitor | 5 (1.3%) | 2 (0.7%) | 3 (3.2%) |
| + Incretin | 17 (4.7%) | 2 (0.7%) | 15 (16.4%) |
| + Insulin | 53 (14.7%) | 51 (18.9%) | 2 (2.1%) |
| + Oral anti-diabetic + Insulin | 5 (1.3%) | 5 (1.8) | 0 (0%) |
| + With other oral anti-diabetic(s) | 3 (.8%) | 3 (1.1%) | 0 (0%) |
| Without metformin | N= 224 | N= 144 | N= 80 |
| Sulfonylurea alone | 57 (25.4%) | 25 (17.3%) | 32 (40%) |
| Glinide alone | 35 (15.6%) | 10 (6.9%) | 25 (31.2%) |
| Thiazolidinedione alone | 7 (3.1%) | 2 (1.3%) | 5 (6.2%) |
| Alpha-glucosidase inhibitor alone | 4 (1.7%) | 2 (1.3%) | 2 (2.5%) |
| Incretin alone | 14 (6.2%) | 2 (1.3%) | 12 (15%) |
| Oral anti-diabetic + insulin | 104 (46.4%) | 100 (69.4%) | 4 (5%) |
| Other oral anti-diabetic combination | 3 (1.3%) | 3 (2.1%) | 0 (0%) |

**Supplementary table 2.** Multivariate regression analysis: age at PD onset is the dependent variable, T2D duration before PD onset, gender and comorbidities are independent variables.

|  | B | SE | β | 95% CI for B | p-value | B | SE | β | 95% CI per B | p-value |
| --- | --- | --- | --- | --- | --- | --- | --- | --- | --- | --- |
| Constant | 61.224 | .301 |  | 60.635, 61.813 | < .001 | 58.719 | 0.334 |  | 58.064, 59.375 | < .001 |
| T2D duration before PD onset (years) | 0.407 | .041 | .114 | 0.326, 0.488 | < .001 | 0.304 | 0.039 | 0.091 | 0.227, 0.381 | < .001 |
| Gender | -1.304 | .264 | -.59 | -1.822, -0.787 | < .001 | -1.36 | 0.264 | -0.062 | -1.879, -0.842 | < .001 |
| Coffee consumption | .74 | .304 | .029 | .143, 1.337 | .015 | 0.373 | 0.32 | 0.014 | -0.254, 0.999 | .244 |
| Smoking | -1.237 | .282 | -.053 | -1.791, -.684684 | < .001 | -1.018 | 0.283 | -0.044 | -1.573, -0.464 | < .001 |
| Heart disease | -- | -- | -- | -- | -- | 4.419 | 0.327 | 0.16 | 3.779, 5.059 | < .001 |
| Hypertension | -- | -- | -- | -- | -- | 4.457 | 0.264 | 0.200 | 3.939, 4.975 | < .001 |
| Stroke | -- | -- | -- | -- | -- | 4.088 | 0.663 | 0.072 | 2.788, 5.387 | < .001 |
| Tumor | -- | -- | -- | -- | -- | 3.785 | 0.405 | 0.109 | 2.990, 4.580 | < .001 |

B: unstandardized regression coefficient; SE: standard error; β: standardized regression coefficient; 95% CI: 95% confidence interval.

**Supplementary table 3.** Multivariate regression analysis: age at PD onset is the dependent variable, T2D duration before PD onset ≤7, T2D duration before PD onset >7, gender and comorbidities are independent variables.

|  | Β | SE | β | 95% CI for B | p-value | Β | SE | β | 95% CI per B | p-value |
| --- | --- | --- | --- | --- | --- | --- | --- | --- | --- | --- |
| Constant | 61.185 | 0.3 |  | 60.597, 61.773 | < .001 | 58.707 | 0.334 |  | 58.051, 59.362 | < .001 |
| T2D duration before PD onset ≤7 | 4.804 | 0.811 | 0.069 | 3.214, 6.394 | < .001 | 2.811 | 0.773 | 0.043 | 1.297, 4.362 | < .001 |
| T2D duration before PD onset >7 | 7.698 | 0.845 | 0.106 | 6.042, 9.354 | < .001 | 5.687 | 0.804 | 0.083 | 4.110, 7.263 | < .001 |
| Gender | -1.326 | 0.264 | -0.06 | -1.843, -0.81 | < .001 | -1.374 | 0.264 | -0.062 | -1.892, -0.856 | < .001 |
| Coffee consumption | 0.715 | 0.304 | 0.028 | 0.119, 1.311 | .019 | 0.374 | 0.319 | 0.014 | -0.252, 0.999 | .242 |
| Smoking | -1.249 | 0.282 | -0.053 | -1.801, -0.696 | < .001 | -1.023 | 0.282 | -0.044 | -1.577, -0.470 | < .001 |
| Heart disease | -- | -- | -- | -- | -- | 4.468 | 0.326 | 0.162 | 3.829, 5.106 | < .001 |
| Hypertension | -- | -- | -- | -- | -- | 4.382 | 0.265 | 0.196 | 3.863, 4.901 | < .001 |
| Stroke | -- | -- | -- | -- | -- | 4.172 | 3.780.658 | 0.074 | 2.883, 5.462 | < .001 |
| Tumor | -- | -- | -- | -- | -- | 3.780 | 0.404 | 0.109 | 2.988, 4.573 | < .001 |

B: unstandardized regression coefficient; SE: standard error; β: standardized regression coefficient; 95% CI: 95% confidence interval.

**Supplementary table 4.** Multivariate regression analysis: age at PD onset as dependent variable, treatment with antidiabetic medication other than metformin before PD onset and comorbidities are independent variables.

|  | Β | SE | β | 95% CI for B | p-value | Β | SE | β | 95% CI per B | p-value |
| --- | --- | --- | --- | --- | --- | --- | --- | --- | --- | --- |
| Constant | 67.059 | .560 |  | 65.957, 68.160 | < .001 | 64.573 | 0.862 |  | 62.877, 66.269 | < .001 |
| Treatment with other antidiabetic medication before PD onset | -.407 | .976 | -.23 | -2.327, 1.513 | .677 | -0.612 | 0.962 | -0.035 | -2.505, 1.281 | .525 |
| Heart disease | -- | -- | -- | -- | -- | 2.912 | 1.003 | 0.157 | 0.938, 4.887 | .004 |
| Hypertension | -- | -- | -- | -- | -- | 1.913 | 0.928 | 0.111 | 0.087, 3.740 | .040 |
| Stroke | -- | -- | -- | -- | -- | 3.083 | 1.705 | 0.098 | -0.272, 6.437 | .072 |
| Tumor | -- | -- | -- | -- | -- | 2.311 | 1.313 | 0.095 | -0.272, 4.894 | .079 |

B: unstandardized regression coefficient; SE: standard error; β: standardized regression coefficient; 95% CI: 95% confidence interval.

**Supplementary table 5.** Multivariate Cox model evaluating the hazard of mortality between PD-preT2D, PD-postT2D and PD-noT2D (number of patients 6930)

|  | B | SE | HR | 95% CI | p-value | B | SE | HR | 95% CI | p-value |
| --- | --- | --- | --- | --- | --- | --- | --- | --- | --- | --- |
| PD-preT2D | .288 | .099 | 1.333 | 1.098, 1.619 | .004 | 0.495 | 0.107 | 1.641 | 1.331, 2.022 | < .001 |
| PD-postT2D | -.345 | .206 | .708 | .473, 1.060 | .094 | -0.151 | 0.245 | 0.860 | 0.532, 1.390 | .538 |
| Male | .339 | .048 | 1.404 | 1.279, 1.541 | < .001 | 0.353 | 0.063 | 1.423 | 1.258, 1.609 | < .001 |
| Age at baseline | .097 | .003 | 1.101 | 1.095, 1.108 | < .001 | 0.107 | 0.004 | 1.113 | 1.104, 1.122 | < .001 |
| PD duration at baseline | .051 | .003 | 1.052 | 1.046, 1.058 | < .001 | 0.061 | 0.004 | 1.062 | 1.054, 1.071 | < .001 |
| Heart disease | -- | -- | -- | -- | -- | -0.283 | 0.065 | 0.754 | 0.664, 0.856 | < .001 |
| Hypertension | -- | -- | -- | -- | -- | 0.124 | 0.065 | 1.132 | 0.996, 1.287 | .057 |
| Stroke | -- | -- | -- | -- | -- | -0.130 | 0.069 | 0.878 | 0.768, 1.004 | .058 |
| Tumor | -- | -- | -- | -- | -- | -0.013 | 0.060 | 0.987 | 0.879, 1.110 | .832 |

The reference group is PD-noT2D.

B: regression coefficient; SE: standard error; HR: hazard ratio; 95% CI: 95% confidence interval.
